# 645. Rapid Versus Conservative Tuberculosis De-isolation Policy Impact Assessment: Province-wide Retrospective Review of 2017-2020, British Columbia, Canada

**DOI:** 10.1093/ofid/ofae631.210

**Published:** 2025-01-29

**Authors:** Othman Zeyad O Alhekail, Navneet Singh, Elisabeth Hansen, James Johnston, Victoria J Cook, William J Connors

**Affiliations:** University of British Columbia, Vancouver, BC, Canada; University of British Columbia, Vancouver, BC, Canada; BC Centre for Disease Control, Vancouver, British Columbia, Canada; University of British Columbia, Vancouver, BC, Canada; University of British Columbia, Vancouver, BC, Canada; University of British Columbia, Vancouver, BC, Canada

## Abstract

**Background:**

Tuberculosis (TB) remains a significant global health threat. Upon diagnosis, patients with pulmonary TB are required to undergo specific isolation precautions to prevent further disease transmission. Current practice typically mandates isolation for at least two weeks post-diagnosis, with extended periods for those found to have positive sputum acid fast bacilli (AFB) smear results. The most recent Canadian Tuberculosis Standards (2022) and other international groups have suggested that the decision to discontinue TB airborne isolation should be made based on length of effective treatment rather than bacteriological endpoint (i.e. Sputum AFB results and culture results).

**Figure:**
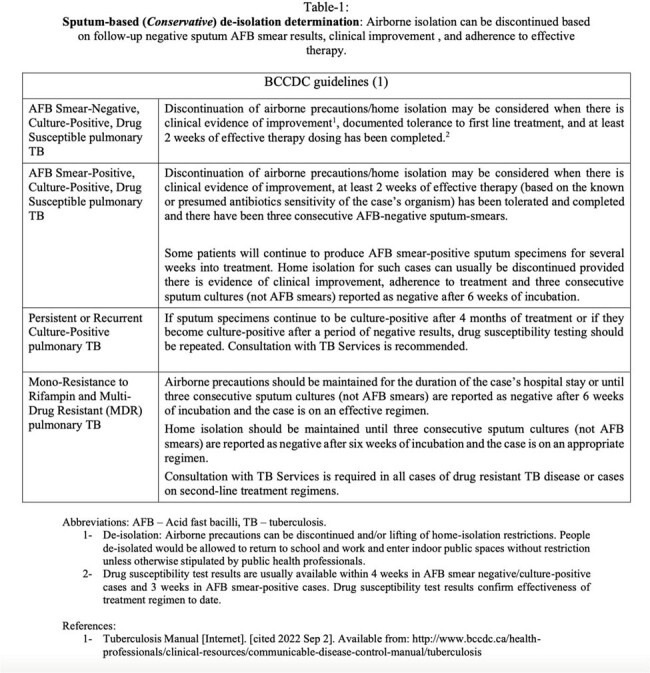

Sputum-based (Conservative) de-isolation determination

**Methods:**

This is a retrospective analysis of newly diagnosed drug susceptible pulmonary TB cases between 2017 and 2020 in British Columbia, Canada. By comparing rapid (defined in Table 1) and conservative (defined in Table 2) isolation determination policies, and estimating recommended isolation periods for each, we aim to quantify isolation periods and assess whether adopting the rapid de-isolation determination protocol would significantly reduce airborne isolation durations.

**Figure:**
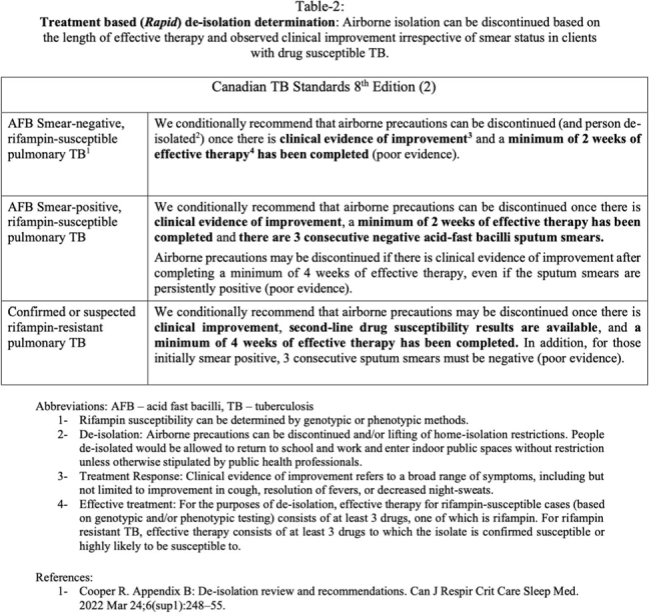

Treatment based (Rapid) de-isolation determination

**Results:**

We identified a total of 952 cases with drug susceptible pulmonary TB. Among the 952, the mean age was 54 years (SD 22). With regards to sputum AFB smear status, 351 (37%) had a negative AFB smear at baseline and 355 (37%) remained sputum AFB smear positive for over 90 days (did not meet conservative de-isolation criteria). Among the remaining 246 cases, 82 converted their AFB smear status to negative within less than 28 days from the first positive sputum AFB smear sample. Based on sputum AFB smear status alone, we found that rapid de-isolation determination would have resulted in a median of 10 (IQR 62) fewer days of isolation.

**Conclusion:**

Adopting a rapid de-isolation determination protocol would result in substantial reductions of isolation durations for patients with drug-susceptible pulmonary TB. Updated evidence-based TB de-isolation guidelines emphasizing effective treatment duration, such as the new recommendation from the National TB Coalition of America, can significantly reduce patients' harm and health system costs of unnecessarily prolonged isolation.

**Disclosures:**

**All Authors**: No reported disclosures

